# Development of hydrogel-based standards and phantoms for non-linear imaging at depth

**DOI:** 10.1117/1.JBO.28.12.126007

**Published:** 2023-12-28

**Authors:** Fizza Haseeb, Konstantinos N. Bourdakos, Ewan Forsyth, Kerry Setchfield, Alistair Gorman, Seshasailam Venkateswaran, Amanda J. Wright, Sumeet Mahajan, Mark Bradley

**Affiliations:** a University of Edinburgh, School of Chemistry, Edinburgh, United Kingdom; b University of Southampton, School of Chemistry, Faculty of Engineering and Physical Sciences, Southampton, United Kingdom; c University of Nottingham, Faculty of Engineering, Optics and Photonics Research Group, Nottingham, United Kingdom; d University of Edinburgh, School of Engineering, Edinburgh, United Kingdom; e Queen Mary University of London, Precision Healthcare University Research Institute, London, United Kingdom

**Keywords:** standards, phantoms, non-linear imaging, hydrogel, depth imaging, axial scaling

## Abstract

**Significance:**

Rapid advances in medical imaging technology, particularly the development of optical systems with non-linear imaging modalities, are boosting deep tissue imaging. The development of reliable standards and phantoms is critical for validation and optimization of these cutting-edge imaging techniques.

**Aim:**

We aim to design and fabricate flexible, multi-layered hydrogel-based optical standards and evaluate advanced optical imaging techniques at depth.

**Approach:**

Standards were made using a robust double-network hydrogel matrix consisting of agarose and polyacrylamide. The materials generated ranged from single layers to more complex constructs consisting of up to seven layers, with modality-specific markers embedded between the layers.

**Results:**

These standards proved useful in the determination of the axial scaling factor for light microscopy and allowed for depth evaluation for different imaging modalities (conventional one-photon excitation fluorescence imaging, two-photon excitation fluorescence imaging, second harmonic generation imaging, and coherent anti-Stokes Raman scattering) achieving actual depths of 1550, 1550, 1240, and 1240  μm, respectively. Once fabricated, the phantoms were found to be stable for many months.

**Conclusions:**

The ability to image at depth, the phantom’s robustness and flexible layered structure, and the ready incorporation of “optical markers” make these ideal depth standards for the validation of a variety of imaging modalities.

## 1 Introduction

Optical imaging technologies provide critical tools for biomedical diagnostics, allowing for non-invasive and real-time detection abilities.^1^ Advances in optical imaging systems, including novel illumination sources, the development of new complementary metal-oxide-semiconductor (CMOS) single-photon avalanche diode (SPAD) detectors, and computational optics, have driven efforts to develop and optimize imaging modalities for deep tissue imaging using longer wavelengths of light.^2^ However, despite the increase in the use of optical imaging methods in clinical practice, published standards for testing, validation, and optimization of new systems are lacking, reducing the speed of translation of optical imaging systems.^3^

Standardized phantoms that can incorporate specific markers or targets for multimodal, non-linear imaging and allow for depth analysis in the hundreds to thousands of micrometer range will prove invaluable in accelerating the progress of deep tissue optical imaging and the development of new imaging methodologies. One way to develop such standards is through laser written fluorescent patterns. This approach has been used to make so-called point spread function (PSF) check slides, which consist of two-dimensional and three-dimensional (3D) patterns fabricated in a polymer substrate and can be used for the rapid and quantitative measure of imaging performance of fluorescence microscopes.^4^ The PSF check slides are created optically and therefore are limited by the working distance restricting the depth of markers. Moreover, the standards are only applicable to fluorescence imaging modalities, whereas the depth standards that we present here can be used for a wide range of non-linear imaging methods, including two-photon excitation fluorescence (2PEF), coherent anti-Stokes Raman scattering (CARS), and second harmonic generation (SHG) imaging. In addition, the depth standards presented in this paper allow for the incorporation of multiple layers of different thicknesses with different scattering and absorption properties. Other studies have employed tissue mimicking standards (also known as phantoms) to evaluate the detectability of imaging targets at depth through the incorporation of channels at millimeter distances within a solid or semi-solid matrix.^5^^–^^7^ Yim et al. designed and developed bilayer phantoms to study the impact of skin phototypes on biomedical optics. Among other applications, the phantoms proved useful for depth analysis during fluorescence imaging. However, these phantoms do not allow for axial resolution in the micrometer range and have a reported shelf life of up to 7 days.^8^ Importantly, there is a need for standards that are robust, stable, and easily transportable to allow for uniform testing and comparison across different systems.^9^

Key features that are typically considered when creating standards are their ease of handling, fabrication, and reproducibility.^10^ Depending upon the application, it may also be necessary to consider biological relevance. Solid-based materials are often preferred due to their robustness and are extensively used for phantom fabrication; however, they are less relevant biologically.^11^ Semi-solid hydrogel-based materials are typically less preferred than solid-based materials as they are prone to water evaporation and are often fragile.^12^^,^^13^ However, hydrogels are better mimics of biological tissues due to their biologically relevant water content and ability to incorporate/form 3D structures resembling the extracellular matrix and tissue architecture.^14^^–^^16^ An added advantage of using water-based semi-solid materials is the ability to optimize the light dose for multiphoton microscopy, a powerful and evolving method for the visualization and analysis of tissues that allow for high-resolution (and ideally deep) optical sectioning with reduced levels of photo damage.^17^ The development and fabrication of hydrogel standards would allow researchers to test a variety of optical setups in a tissue-like environment.^18^ An approach to overcome the well-known limitations of hydrogels is reinforcing the traditional single network hydrogel through the incorporation of a second network (making a so-called double network), making the gels robust and tough.^19^

**Fig. 1:**
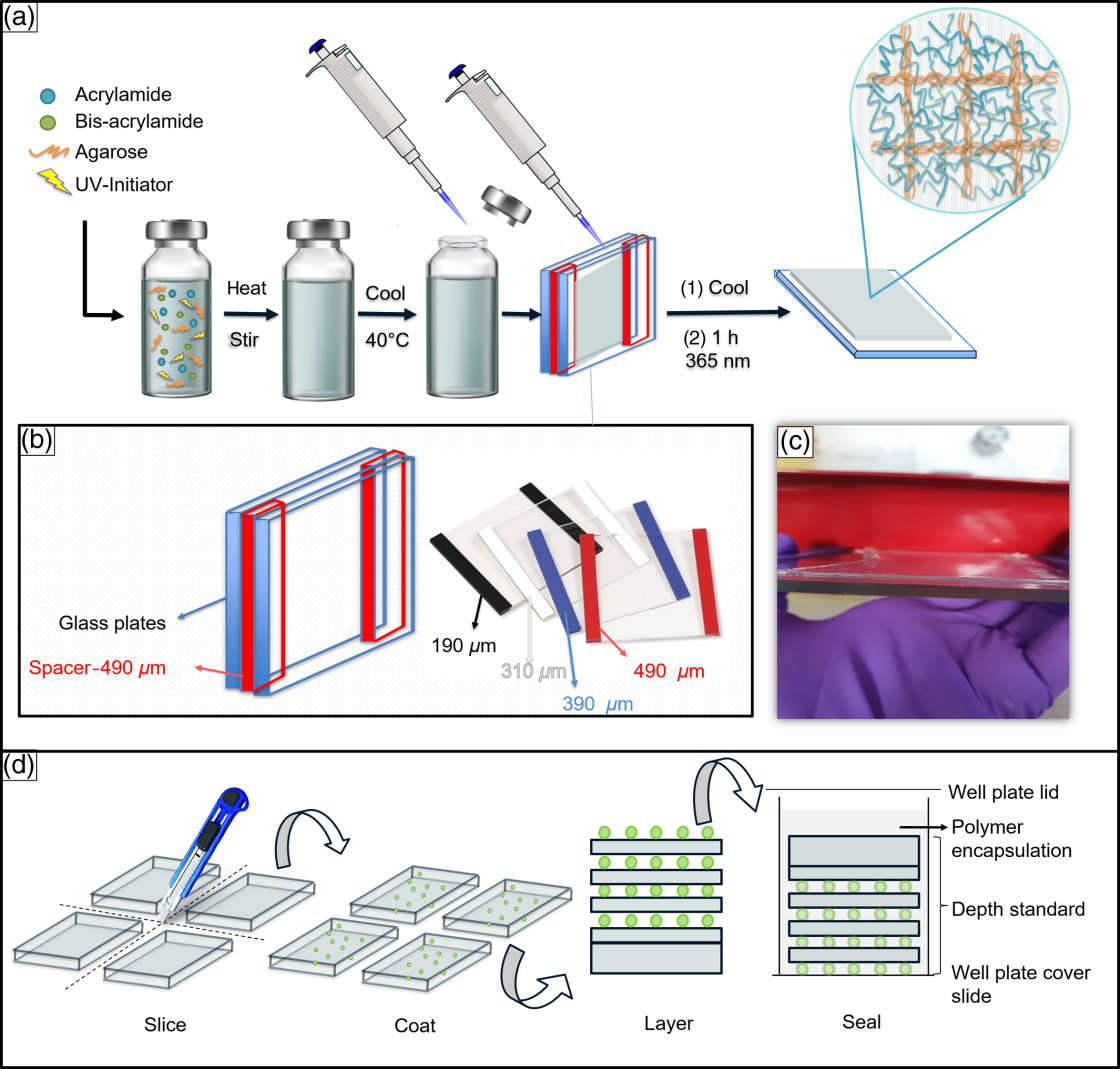
(a) One pot method for the fabrication of the double network hydrogels. Agarose, acrylamide, N, N′-methylene-bis-acrylamide, and the ultraviolet (UV) activated initiator were mixed in water, heated, cooled, and UV cured to obtain the double network hydrogel. (b) Casting mold assembly comprising two glass plates (treated with 1H,1H,2H,2H-Perfluorooctyldimethylchlorosilane) using a variety of spacers of differing thicknesses; (c) 490  μm double network hydrogel mounted on a glass plate ; (d) the 4 stages involved in the fabrication of the depth phantoms, i.e., slicing, coating with modality specific markers, layering, and sealing.

In this paper, we demonstrate the utility of a flexible, inexpensive, multilayer depth standard (or depth phantom) with the use of double network hydrogel-based matrices generated using two commonly applied hydrogels, agarose, and polyacrylamide. Using a rapid two-step but “one-pot”^20^ fabrication method, the gels were cast and fabricated as thin double network layers from 190 to 2000  μm in thickness [Figs. 1(a)–1(c)]. A variety of depth phantoms were then fabricated as multi-layered stacks of hydrogels, with imaging modality-specific markers incorporated between the layers [Fig. 1(d)]. Modality-specific markers included ${\mathrm{BaTiO}}_{3}$ for SHG,^21^ fluorescent-silica (FS) beads for one-photon excitation fluorescence (1PEF) and 2PEF,^22^^,^^23^ and polystyrene (PS) beads for CARS.^24^ The particles were selected based on their accessibility and sensitivity to the respective imaging modalities. Sealing using polydimethylsiloxane (PDMS) produced hydrogel standards that were protected from dehydration and were stable for more than 2 months, thus reducing the barriers to their adoption across the research community. Here, these depth phantoms were evaluated using 1PEF, 2PEF, coherent anti-Stokes Raman imaging, and SHG imaging.

## 2 Materials

FS beads were prepared as previously described.^22^ PS beads (10  μm) and ${\mathrm{BaTiO}}_{3}$ were purchased from Rapp Polymere GmbH (Catalog number: HM1502) and Merck Life Sciences United Kingdom (Catalog number: 208108-500G). 2-hydroxy-4’-(2-hydroxyethoxy)-2-methylpropiophenone (Irgacure 2959) was purchased from Fluorchem, and high-pressure liquid chromatography (HPLC) grade water was from Fisher Scientific; all other chemicals were from Sigma unless specified. The UV light source used for all polymerization work was a UVP (model CL-1000, 365 nm, 8 Watt, $1000\;\mathrm{mJ}\;{\mathrm{cm}}^{-2}$).

## 3 Methods

### 3.1 Fabrication of Depth Standards

#### 3.1.1 Functionalization of glass plates with a fluorosilane

1H,1H,2H,2H-Perfluorooctyldimethylchlorosilane (5% w/v in Toluene, 5 mL) was dispensed into a clean glass desiccator. Glass plates (75×50  mm, Corning^®^, 2947-75×50) were placed on a wire rack inside the desiccator, a vacuum was applied (5 min), and the desiccator was sealed for 12 h. The glass plates were then washed with acetone (2×5  mL) and used the same day.

#### 3.1.2 Preparation of precursor solution

Acrylamide (1.78 g), N,N′-methylene-bis-acrylamide (1.8 mL, 1% w/v in water), Irgacure 2959 (56 mg), Agarose (200 mg), and water (8.2 mL, HPLC grade) were added into a glass vial (Biotage^®^ 10 to 20 mL, Part No. 354833), flushed with nitrogen (30 min), sealed, and heated in an oil bath to 90°C to dissolve the agarose.

#### 3.1.3 Fabrication of the double-network gels of defined thickness

To prepare the double-network gels (thickness>200  μm), two plastic spacers (RS PRO shim kit, RS Stock No.: 681-407 with a thickness of 310, 390, 490, 760  μm and Fisherbrand™ Bonded Spacers, Product Code: 11807653 with thickness 570  μm) were placed between two fluorosilane coated glass plates. To prepare the 2 mm support layer, fluoro-silane coated glass plates with 1.0 mm Integrated Spacers (Bio-rad Mini-PROTEAN^®^ Spacer Plates, Catalog number: 1653311) were set up with spacers facing inward to create a gap of 2 mm. The gap between the glass slides on three sides was sealed using autoclave tape, and the precursor solution (∼90°C) was dispensed through the open top until full. Double-network gels of <200  μm were prepared by placing a few drops of the precursor solution (∼90°C) onto one fluoro-silane coated glass plate. Spacers (RS PRO shim kit, RS Stock No.: 681-407) were positioned on either side of the drops prior to placing another fluoro-silanised glass plate on top. The space between the two plates was visually inspected to ensure no air gaps, and these were then sealed on all four sides. The sealed assembly was allowed to cool to room temperature and UV treated for 60 min.

#### 3.1.4 Fabrication of single-layered and multi-layered depth phantoms

Suspensions of signaling markers consisting of PS beads, FS beads, or ${\mathrm{BaTiO}}_{3}$ were prepared by adding 0.2 mg in 200  μL of ethanol in 1.5 mL Eppendorf tubes. Suspended PS beads were sonicated for 30 min to achieve good dispersion. The three suspensions were then spin-coated (a SCS 6800 series spin coater, 1000 rpm, 5 s) onto 3 mm diameter circular glass coverslips using 5  μL of suspension per coverslip, followed by drying in a desiccator for 30 min (under vacuum) or at room temperature for 12 h.

For single-layered phantoms, spin-coated coverslips were placed in contact with both sides of a 6  mm×6  mm section of a double network hydrogel of defined thickness, and the signaling markers were transferred from the coverslip onto the gel. For multi-layered phantoms, coated hydrogel layers were stacked as required. Both the single- and multi-layered phantoms were placed onto a 2 mm thick double network hydrogel support layer. The assembly was placed into a 4-well plate (Ibidi Cat.No: 80426) with the 2 mm support layer uppermost and gently pressed to ensure good contact with the surface. Vinyl terminated PDMS (SYLGARD™ 184 kit, Dow Corning) and curing agent (part of the kit) were mixed (9 g: 1 g) in a glass beaker (50 mL), heated (80°C, 3 min) in an air-assisted oven, cooled to 25°C and poured into the wells containing the single or multi-layered phantoms until they were completely immersed in the PDMS mixture. The well plates were then placed in a glass desiccator, a vacuum was applied to remove air bubbles from the PDMS mixture, and they were allowed to cure by incubating at room temperature for 24 h.

### 3.2 Refractive Index Measurement of Double Network Hydrogel

Refractive index measurements of double network hydrogels were made using a refractometer (Bellingham + Stanley, A180032). Measurements were made at room temperature (20°C) using a sodium lamp of wavelength 589 nm.

### 3.3 Characterization of the Thickness of Single and Layered Phantoms

The depths of hydrogels of different thickness (spacer thickness 190, 310, 390, 490, 570, or 760  μm) were measured using (1) digital Vernier calipers, (2) bright-field microscopy, and (3) fluorescence microscopy.

Vernier caliper measurements were taken using a Duratool. D03196. For bright-field microscopy, the individual gel layers were sectioned using a razor blade, the cross-section was viewed using a Leica microscope (DMC6200, HC PL FLUOTAR, 10×), and the thickness of the gels was measured [using Leica Application Suite X software (3.4.2.18368)] from the distance traversed by the microscope stage when moved from one edge of the gel to the other.

For fluorescence microscopy thickness analysis, two microscopes were used.

1. Zeiss Axiovert 200M, (Plan-Neofluar, 20×, λex=544  nm, λem=570  nm) and
2. Nikon Eclipse Ti-S microscope (Nikon S-Plan Fluor ELWD, 40×, λex=488  nm, λem=510 to 530 nm).

Single- and multi-layered phantoms were prepared with FS beads, and the thickness of the hydrogel layer (n=6 for each spacer thickness) was determined using the z-stack feature of the microscope, measuring the distance between the image plane of the beads at either side of the test gel.

### 3.4 CARS, SHG, and 2PEF Characterization of the Multi-Layered Phantoms

A custom-built, multimodal laser scanning microscope was used to acquire images (using ScanImage^®^,^25^ Vidrio Technologies LLC) with coherent anti-Stokes Raman scattering (CARS), SHG, and 2PEF. For CARS imaging, the Stokes beam was the fundamental of a fiber laser (1031 nm, Emerald Engine, APE), and the pump beam was generated by an optical parametric oscillator (OPO) (650 to 950 nm, Levante Emerald, APE GmbH), which was synchronously pumped by the second harmonic (515.5 nm) of the fiber laser. The repetition rate and pulse width for both beams were 80 MHz and 2 ps, respectively. The two beams were spatiotemporally overlapped and made collinear before being coupled (through a galvanometric scanner) into an inverted microscope (Nikon Ti-E) and focused through a 20×/0.75 NA Nikon objective on the sample. All of the signals (CARS, SHG, 2PEF) were collected through the same objective in a back-scattering geometry (epi-detection), and through the appropriate dichroic and band-pass filters, and were delivered to three Hamamatsu photomultiplier tubes (PMTs). Two PMTs were used for signal acquisition (H10722-20, Hamamatsu photonics), one each for the CARS and 2PEF channels, and one PMT (H10722-210 Hamamatsu photonics) was used in the SHG channel. All of the images were acquired with a pixel dwell time of 8  μs, with a 512×512  pixel resolution, covering a field of view of 250×250  μm, and they were processed with FIJI (ImageJ, Wayne Rasband and contributors, National Institute of Health, United States).^26^

## 4 Results and Discussion

### 4.1 Depth Characterization Reveals Consistent Thickness of Single-Layered and Multi-Layered Phantoms

The axial measurements of single layers of double-network gels made using Vernier calipers, bright field microscopy (cross-section measurements of the gels), and fluorescence microscopy showed repeatability in the construction of hydrogel layers of defined thicknesses (see Table 1). Thicknesses measured via digital Vernier calipers and bright-field imaging (10×, DMC6200, HC PL FLUOTAR, Leica microscope) of the cross-section of the gels were consistent and agreed with spacer thickness (see Table 1).

**Table 1:** Repeatability in fabrication of double network hydrogels of defined thicknesses assessed using Vernier calipers, bright-field imaging, and fluorescence microscopy. Correspondence is the thickness of the hydrogel divided by the thickness of the spacers (×100). M, measurement; C, correspondence.

Spacers	Hydrogel thickness measurement					
Vernier caliper	Vernier caliper		Bright field		Fluorescence	
M (μm)	M (μm)	C (%)	M (μm)	C (%)	M (μm)	C (%)
190	190	100	174 ± 6	91	124 ± 3	65
310	310	100	304 ± 8	98	218 ± 6	70
390	370	95	365 ± 8	94	260 ± 4	67
490	490	100	479 ± 5	98	331 ± 8	68
570	570	100	561 ± 11	98	390 ± 10	68
760	720	95	717 ± 4	94	505 ± 3	66

**Fig. 2:**
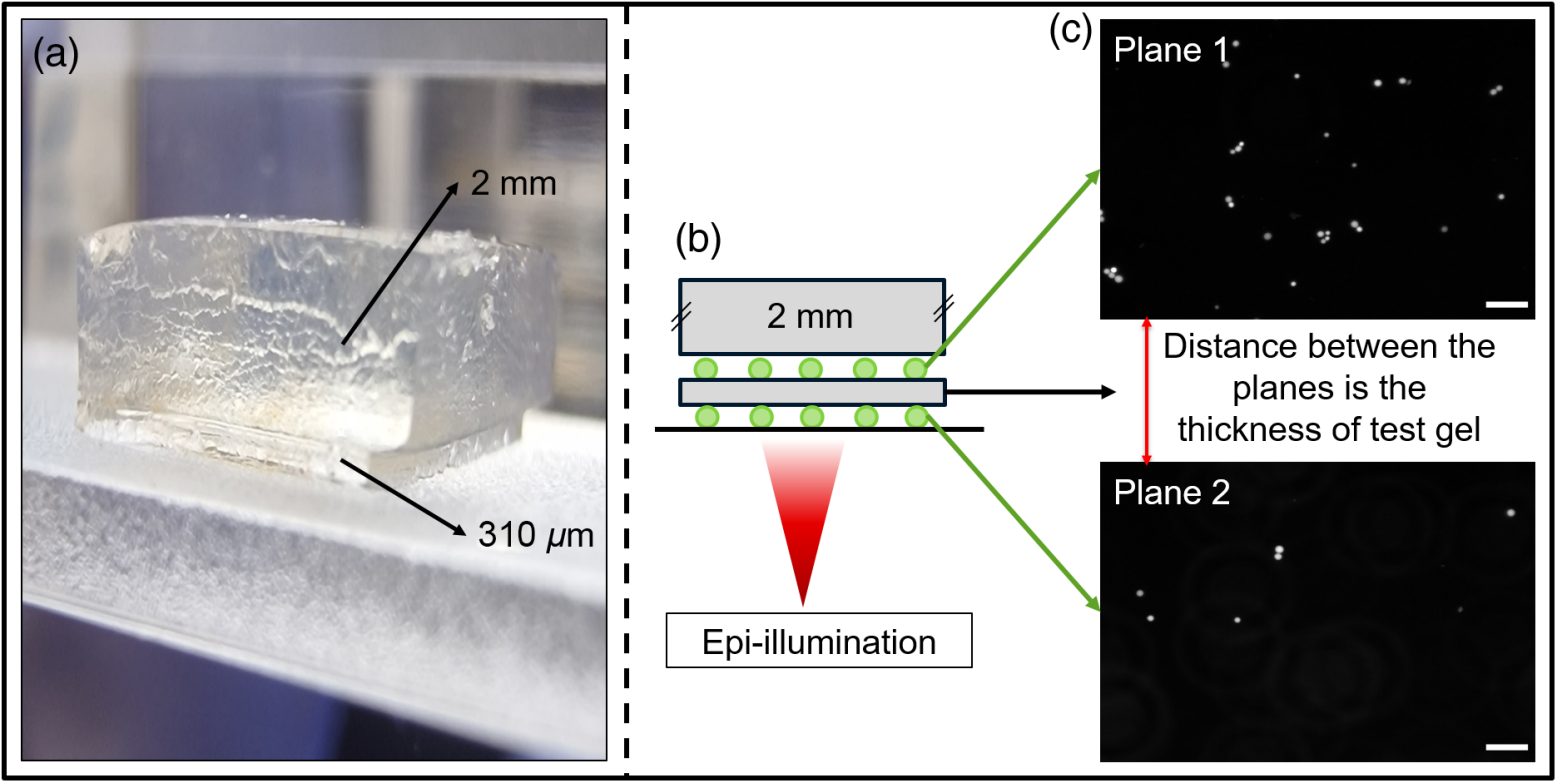
Measurement of the thickness of a gel layer. (a) Gel sample used for thickness analysis with a lower layer of 310  μm (test layer) capped with a 2 mm support layer. (b) Imaging a single gel layer (310  μm) coated with FS beads on both surfaces and then capped with a support layer of gel (2 mm). (c) Microscopy images of the marker layers on the top and bottom of the 310  μm gel. The distance traversed by the microscope stage between the image planes of the beads gives the apparent thickness of the gel. The scale bar represents 50  μm.

Vernier caliper measurements agreed with the bright field measurements (Table 1, Fig. S1 in the Supplementary Material); however, these measurements did have an error associated with the compression of gels. However, the thicknesses were significantly different from the measurements made using fluorescence microscopy (see Table 1). The experimental setup for fluorescence microscopy is illustrated in Fig. 2 with the test gel sandwiched between two layers of FS beads, and the thickness was determined from the axial (z) distance traversed between the two adjacent image planes containing fluorescent beads (the beads were monodispersed and 10  μm in diameter). For each measurement, six samples were analyzed, and the values are presented in Table 1 along with the standard deviation. This apparent difference in axial measurements is expected due to refractive index mismatch (the refractive index of hydrogel is 1.37 and air is 1.0003^27^). Thus, when measured through a microscope, it will result in an apparent depth/measurement depth that is smaller than the actual depth.^28^^,^^29^ The bright field and fluorescence measurements differed by a factor of 1.42±0.02, and this factor here is referred to as the correction factor. This value was validated (using Zemax OpticStudio 21.3.) with raytracing to determine the relationship between the axial translation and the sample thickness. Considering the numerical aperture of the lens used (0.4) and the refractive index of the hydrogel (1.37), the correction factor calculated was 1.41 for gels ranging in size from 100 to 500  μm (see Table S1 in the Supplementary Material).

**Fig. 3:**
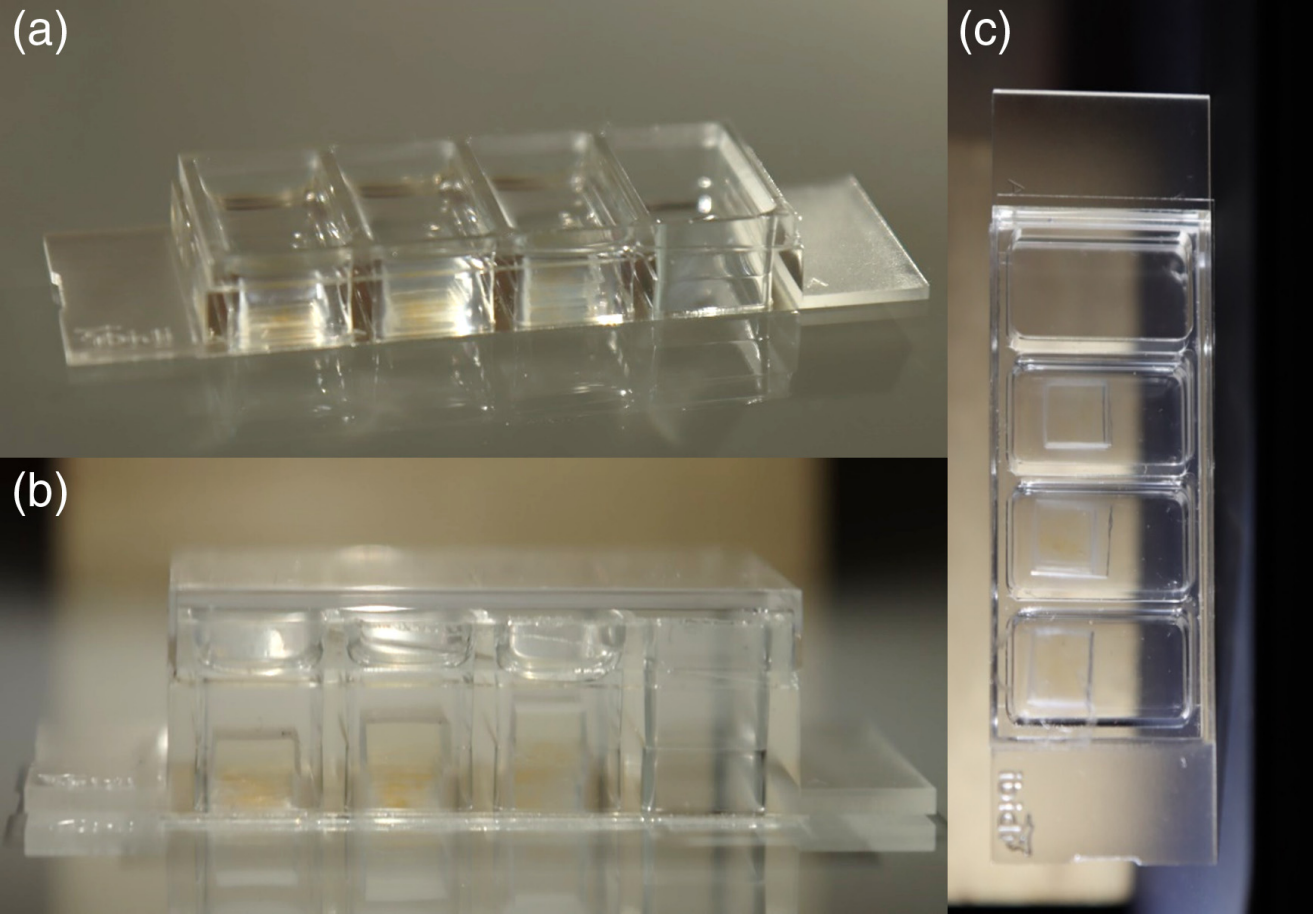
Images of well plates containing the depth standards. (a), (b) Side views and (c) bottom view of the fabricated standards. Well plate outer dimensions (w×l) 25.5  mm×75.5  mm.

**Fig. 4:**
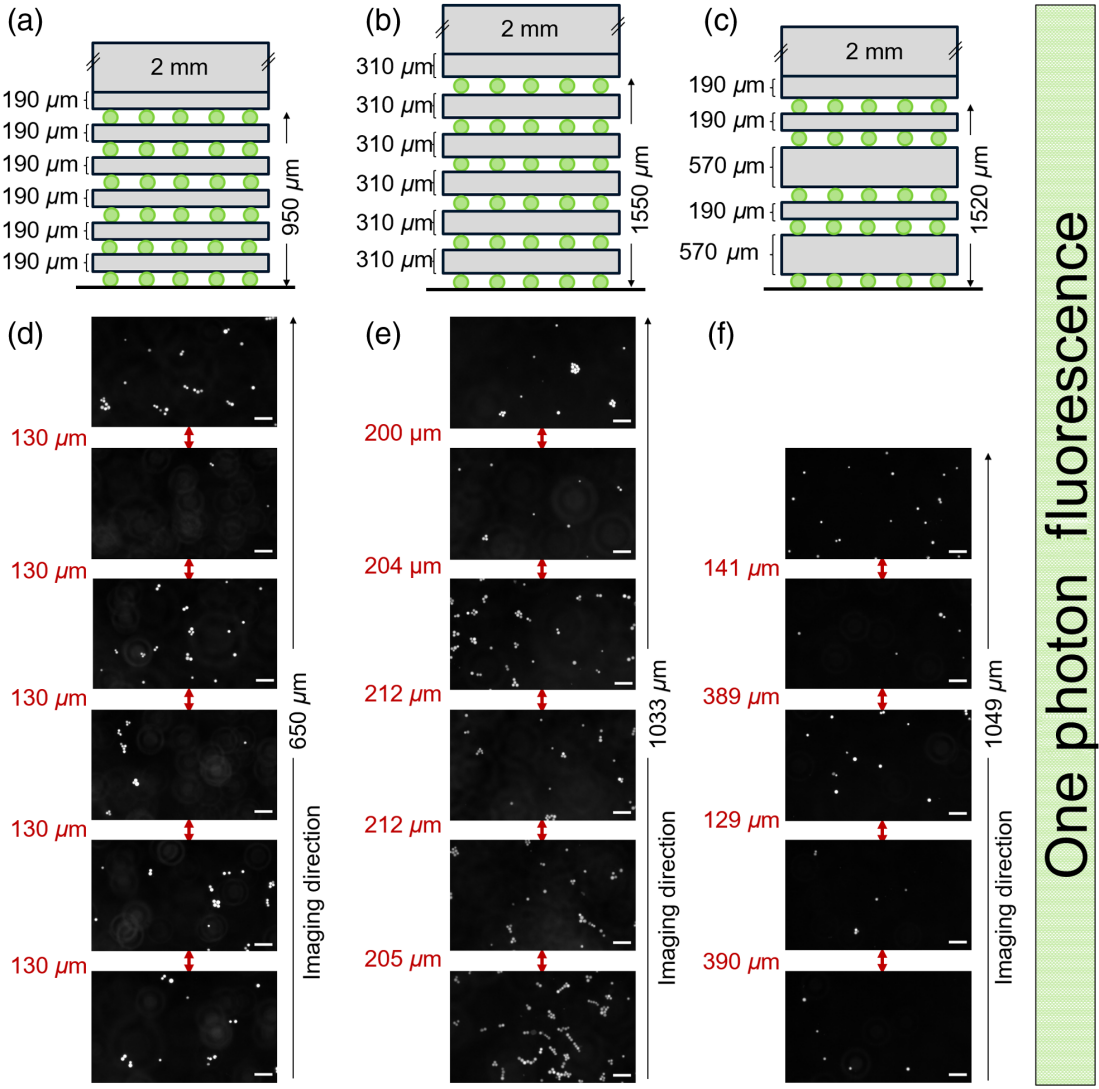
Measurements of the thickness of the gel layers in the multi-layered phantoms showing the expected thickness from the fabrication process. (a)–(c) The design of the depth standards for the testing of optical systems. Depth standards (a) and (b) were made of 6 layers of signaling markers sandwiching 5 layers of hydrogels of defined thicknesses giving overall heights of 950 and 1550  μm. Depth standard (c) was made of 5 layers of signaling markers sandwiching 4 layers of hydrogels of defined thicknesses giving overall heights of 1520  μm. (d)–(f) Images of signaling markers in each sample, generated via 1PEF with the separation between each layer in μm, are given in red. The scale bar represents 50  μm.

Following the success of these initial designs and thickness measurements, 3 new multi-layered depth standards were prepared, with overall actual depths of 950, 1550, and 1520  μm and with the thicknesses of the individual layers varying (190, 310, and 570  μm) (see Figs. 3 and 4).

The thickness measurements of these multi-layered phantoms were consistent using this technique (Fig. 4), showing apparent depths of 650, 1033, and 1049  μm for the 950, 1550, and 1520  μm standards, respectively. Measurements done on three different samples of the same design showed that standards were easily reproducible (see Fig. S2 in the Supplementary Material). When the measurements were made using a different fluorescence microscope (Nikon Eclipse Ti-S microscope, Nikon S-Plan Fluor ELWD, 40×), the apparent depth in the 3 designs was 650, 875 (up to the 5th layer of marker), and 997  μm, demonstrating the robustness of the standards (see Fig. S3 in the Supplementary Material). The analysis of the temporal stability of standards over 60 days showed (Table 2) the stability of the gels once sealed. The robustness, stability of the standards, and compact design suggest easy handling and transport across systems and institutions that would allow for consensus and standardization in measurement.

**Table 2:** Depth measurements (in μm) of the multi-layered phantoms after 0, 46, and 58 days as measured via fluorescence (one-photon) microscopy.

Design 1			Design 2			Design 3		
Day 0	Day 46	Day 58	Day 0	Day 46	Day 58	Day 0	Day 46	Day 58
130	126	125	200	189	186	389	375	363
130	132	128	204	206	186	141	125	124
130	125	125	212	230	207	390	367	370
130	124	122	212	250	198	121	130	128
131	143	145	205	No marker seen	203			

### 4.2 Depth Penetration Evaluation of Non-Linear Imaging modalities

**Fig. 5:**
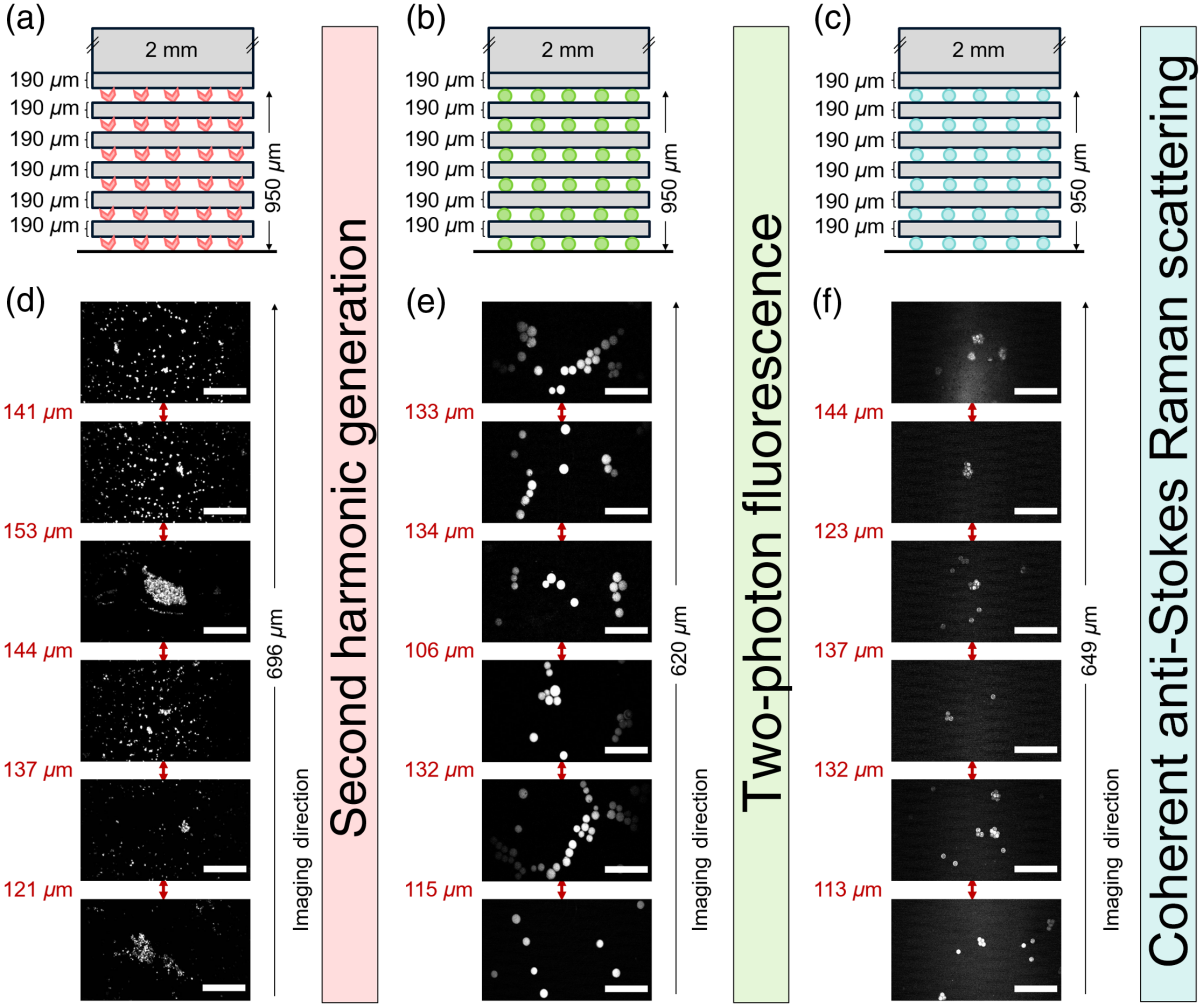
Analysis of depth standard design 1 on multimodal imaging systems. (a)–(c) Illustration of depth standards incorporating “modality markers” BaTiO3 (red), FS beads (green), and PS beads (blue) and imaged via SHG, 2PEF, and coherent anti-Stokes Raman scattering, respectively. Each depth standard had 6 layers of signaling markers sandwiching 5 layers of hydrogels of defined thicknesses (190  μm) giving an overall height of 950  μm. (d)–(f) SHG, 2PEF, and CARS imaging up to depths of 696, 620, and 649  μm, respectively. The microscopy images represent the marker layers, and the distances between each layer are given in red. The scale bar represents 50  μm.

**Fig. 6:**
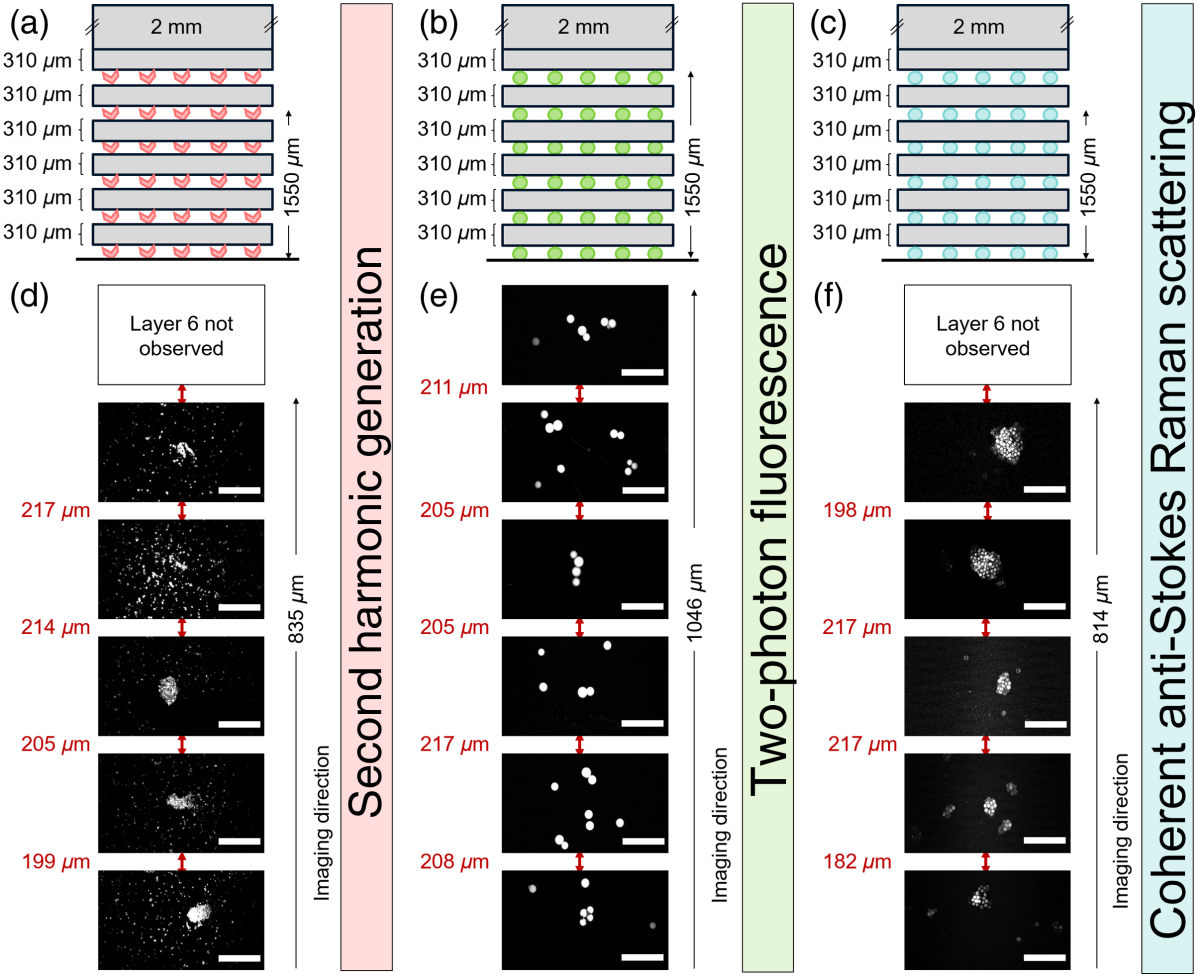
Analysis of depth standard design 2 on multimodal imaging systems. (a)–(c) Depth standards incorporating BaTiO3 (red), FS beads (green), and PS beads (blue) imaged via SHG, 2PEF, and coherent anti-Stokes Raman scattering, respectively. Each depth standard had 6 layers of signaling markers (BaTiO3/FS beads/PS beads) sandwiching 5 layers of hydrogels of defined thicknesses (310  μm) giving an overall height of 1550  μm. (d)–(f) SHG, 2PEF, and CARS imaging up to depths of 835, 1046, and 814  μm, respectively. The microscopy images represent the marker layers, and the distances between each layer are given in red. The scale bar represents 50  μm.

Three sets of standards were designed for the various non-linear imaging modalities 2PEF, SHG, and CARS (see Figs. 5 and 6). Each set had 2 constructs, one with 190  μm hydrogel layers and the other with 310  μm hydrogel layers between the signaling markers. For each imaging modality, a suitable marker was selected and incorporated into the depth standard, e.g., FS beads for 2PEF, PS beads for CARS, and ${\mathrm{BaTiO}}_{3}$ nanocrystals for SHG imaging.

The samples were imaged with a custom-built, multimodal laser scanning microscope (described in Sec. 3). In more detail, the C–H stretching mode at $2845\;{\mathrm{cm}}^{-1}$ was used for CARS imaging of the PS beads by tuning the OPO at 797 nm and collecting the anti-Stokes at 650 nm (Figs. 5(c) and 6(c)]. Furthermore, the ${\mathrm{BaTiO}}_{3}$ crystals were imaged via the means of SHG of the OPO beam collecting the corresponding signal around 400 nm [Figs. 5(a) and 6(a)]. Finally, the fluorescent beads were imaged with 2PEF using the degenerate two-photon excitation of both beams (with wavelengths of 1031 and 797 nm, respectively), as well as the non-degenerate two-photon excitation of their combination as verified by the partial delay dependence of the signal, which was collected in the spectral range between 530 and 570 nm [Figs. 5(b) and 6(b)]. It should be noted here that no focus-aiding technology was used for acquiring the images of the signaling marker; instead, images were acquired by adjusting the objective to a position at which the maximum contrast/maximum sharpness could be seen by eyes and the diameter of the beads could be determined.

Importantly, all 6 layers of “signaling markers” were detected when imaging through the samples separated by 190  μm hydrogel layers going up to apparent depths of 696, 620, and 649  μm using SHG, 2PEF, and CARS, respectively (the actual depth corrected achieved was 950  μm) (Fig. 5). However, in samples constructed with the 310  μm hydrogel layers, 6 layers of signaling markers could only be imaged using 2PEF, which reached depths of 1046  μm (the actual corrected depth achieved was 1550  μm). By comparison, using SHG and CARS imaging, only 5 layers were imaged, reaching depths of 835 and 814  μm, respectively (the actual depth achieved was 1240  μm) (Fig. 6) (here the depth evaluation was limited by the working distance of the microscope objective, which was 1000  μm). It should be noted that, as expected, the incident laser power needed to be increased when imaging depths>500  μm to generate sufficient signal (see Table S2 in the Supplementary Material).

For single and multiphoton microscopy, a good signal to background ratio was achieved. Agarose and polyacrylamide gels have been extensively characterized using ultra-violet-visible and near infra-red (UV-Vis-NIR) and Raman spectroscopy,^30^^–^^33^ and polyacrylamide gels are known to exhibit minimal autofluorescence in a limited wavelength region (350 to 500 nm), which was not used in this study, whereas agarose gels are generally considered non-fluorescent.^34^^–^^37^ For CARS imaging, only the symmetric methylene (CH2) stretching vibration was probed at $2845\;{\mathrm{cm}}^{-1}$. CARS signal depends quadratically on the number of oscillators in the focal volume, hence, the signal from the PS beads was much higher than the negligible background from the polymers in the hydrogels (which contain ∼80% water).

## 5 Conclusion

The reliability and repeatability of biophotonic instrumentation is often hampered by a lack of recognized standards and standardized phantoms suited for technical evaluation, device building, and optimization. Often, the preparation of standards/phantoms for the routine calibration of optical systems can be a time-consuming task and subject to person-to-person variability. To enable the routine validation of new biomedical imaging systems and aid the process of deep tissue imaging, we developed stable, robust, and highly reproducible depth standards using a biologically relevant hydrogel-based matrix. These phantoms, once prepared, can be stored at room temperature and utilized for on-the-spot verification and testing of optical systems. The phantoms proved to be powerful tools for depth evaluation of various imaging modalities, including 1PEF, 2PEF, SHG, and CARS, allowing for imaging depths of 1550, 1550, 1240, and 1240  μm, respectively. Moreover, our depth standards can potentially be used to calibrate the axial positioning and movement of microscope systems providing a much-needed step toward their standardization. These standards have potential to be used in adaptive optics for the correction of optical aberrations at depth and allow for the optimization and development of imaging systems with micrometer scale resolution, helping to improve new imaging modalities and systems. The ability to incorporate “on-demand” markers, in particular, is a huge advantage for multimodal imaging systems such as those used in this work. The standards presented here are versatile, allowing a variety of signaling markers to be added, at desired depths, to provide targets of interest for multiple imaging modalities. In addition, owing to their controllable water content, these phantoms will prove useful for safety evaluation of near infrared light in biomedical imaging as water has absorption peaks within this region. Our standards can be modified by the incorporation of additives to further mimic the optical properties of tissues, thereby allowing for the evaluation of new imaging modalities that are becoming increasingly invaluable to a wide variety of research areas.

## Data, Materials, and Code Availability

All relevant data, materials (see Sec. 2), and codes are included in the paper and Supplementary Material.
