# Supplementary material for: Development of hydrogel-based standards and phantoms for non-linear imaging at depth
Source: J Biomed Opt. 2023 Dec 28;28(12):126007. doi: 10.1117/1.JBO.28.12.126007 (PMC10753126; doi:10.1117/1.JBO.28.12.126007)
Supplement: Supplementary file 1 [file JBO_028_126007_SD001.pdf]

Development of hydrogel-based standards and phantoms for non-linear imaging at depth

Fizza Haseeb[a], Konstantinos N. Bourdakos[d], Ewan Forsyth[a], Kerry Setchfield[c], Alistair Gorman[b], Seshasailam Venkateswaran[e], Amanda J. Wright[c], Sumeet Mahajan[d], Mark Bradley[e*]

[a]School of Chemistry, University of Edinburgh, David Brewster Road, EH9 3FJ Edinburgh, UK
[b]School of Engineering, University of Edinburgh, Alexander Crum Brown Road, Edinburgh EH9 3FF
[c]Optics and Photonics Research Group, Faculty of Engineering, University of Nottingham, Nottingham, NG7 2RD
[d]School of Chemistry, Faculty of Engineering and Physical Sciences, University of Southampton, SO17 1BJ Southampton, UK.
[e]Precision Healthcare University Research Institute, Queen Mary University of London, Whitechapel, Empire House, London E1 1HH, UK

Supplementary Data

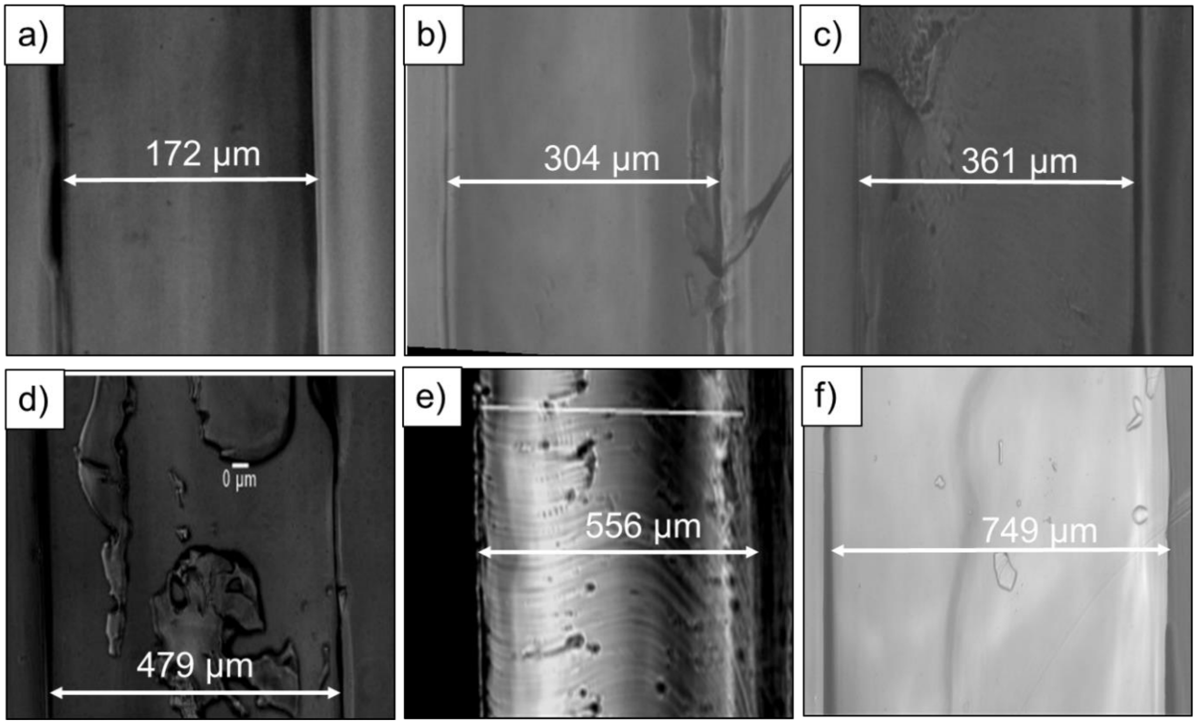

Supplementary Figure 1. Thickness measured from the bright-field images of the double-network gels prepared using the plastic spacers with thicknesses of; a) 190 µm, b) 310 µm, c) 390 µm, d) 490 µm, e) 570 µm and f) 760 µm

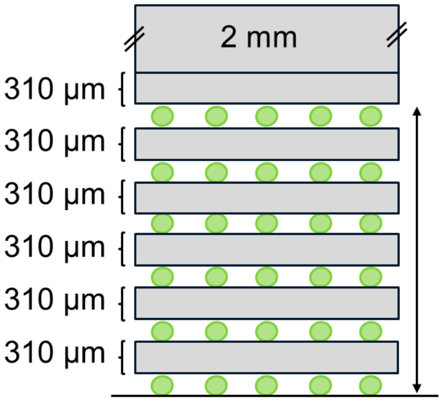

Sample 1 (µm)	Sample 2 (µm)	Sample 3 (µm)
200	202	212
204	216	225
212	204	208
212	215	211
205	202	229

Supplementary Figure 2. Repeatability in the construction of multi-layered standards. Left: Sample design consisting of 6 marker layers sandwiched between layers of hydrogel of thickness 310 µm. Right: Table showing measurements on 3 different multi-layered samples of the same design.

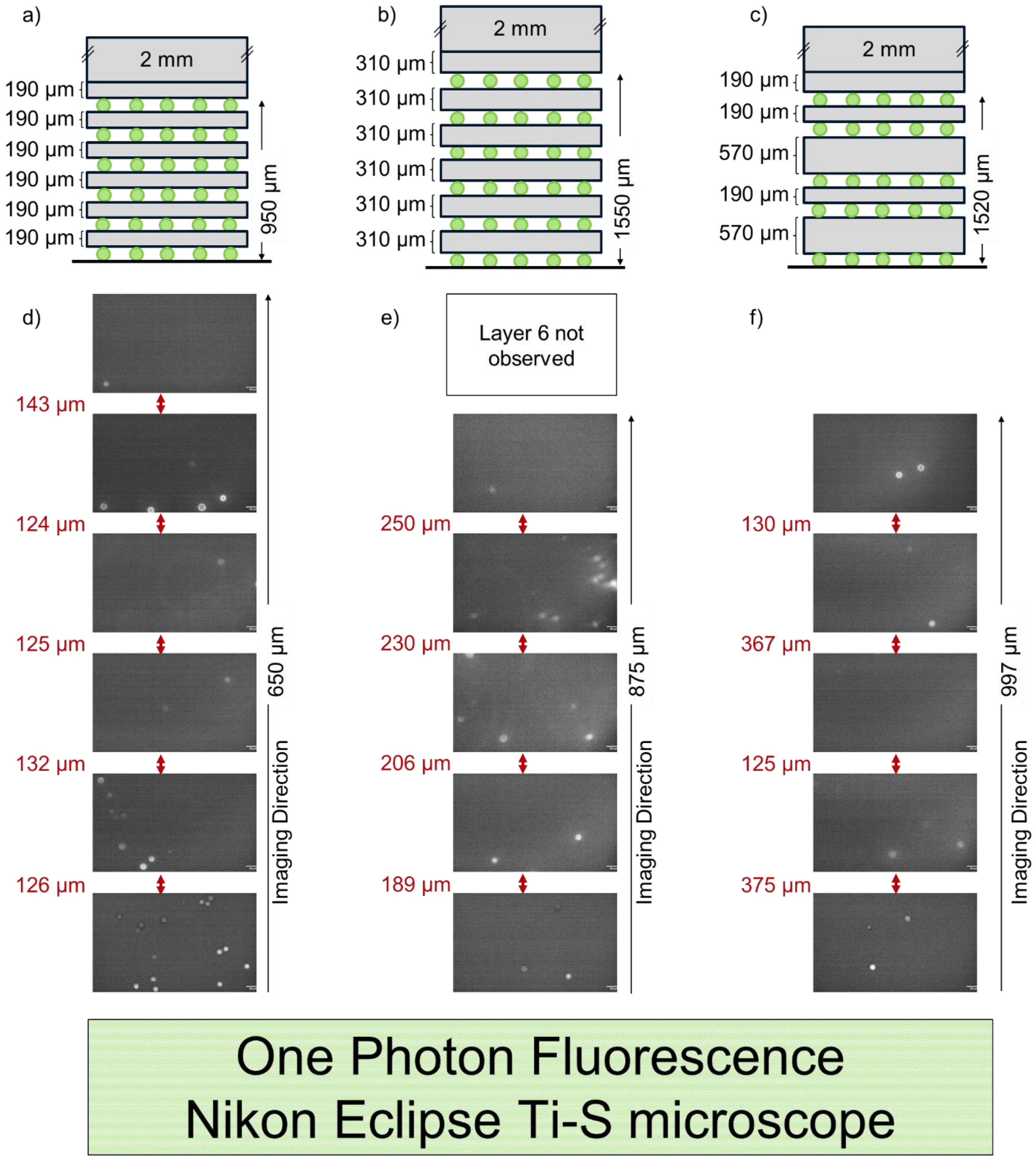

Supplementary Figure 3. Depth standards design and one-photon excitation fluorescence imaging (Nikon Eclipse Ti-S microscope, Nikon S-Plan Fluor ELWD, 40x, λex = 488nm). (a), (b) and (c) the designs of the depth standards used for imaging. (d), (e) and (f) the imaging done on designs a, b, and c, respectively.

The scale bar represents 25 µm.

Supplementary Table 1. Determination of correction factor for axial scaling in light microscopy using Zemax OpticStudio.

Sample depth (mm)	Axial shift (mm)	Ratio (Correction factor)
0.1	0.0709	1.4113
0.2	0.1418	1.4105
0.3	0.2127	1.4103
0.4	0.2836	1.4102
0.5	0.3546	1.4101

Supplementary Table 2. Pump and stokes beam laser powers for multiphoton microscopy.

**Layers**	**Manuscript Figure 5**		**Manuscript Figure 6**	
	stokes (1031 nm) power (mW)	pump (797.2 nm) power (mW)	stokes (1031 nm) power (mW)	stokes (1031 nm) power (mW)
SHG 1st Layer	N/A	9.2	N/A	9.2
SHG 2nd Layer	N/A	18.7	N/A	18.7
SHG 3rd Layer	N/A	18.7	N/A	18.7
SHG 4th Layer	N/A	18.7	N/A	28.5
SHG 5th Layer	N/A	28.5	N/A	70
SHG 6th Layer	N/A	28.5	N/A	N/A
TPEF 1st Layer	75	18.4	25	9.2
TPEF 2nd Layer	75	18.4	25	9.2
TPEF 3rd Layer	75	71	75	18.4
TPEF 4th Layer	75	71	75	18.4
TPEF 5th Layer	75	71	75	28.5
TPEF 6th Layer	75	71	75	28.5
CARS 1st Layer	25	17.8	75	28.5
CARS 2nd Layer	75	28.5	75	28.5
CARS 3rd Layer	75	28.5	75	28.5
CARS 4th Layer	75	28.5	75	28.5
CARS 5th Layer	75	28.5	75	28.5
CARS 6th Layer	112	46	N/A	N/A
